# Secreted Phospholipase A2 Involvement in Neurodegeneration: Differential Testing of Prosurvival and Anti-Inflammatory Effects of Enzyme Inhibition

**DOI:** 10.1371/journal.pone.0039257

**Published:** 2012-06-15

**Authors:** Shuyan Chen, Lihua Yao, Timothy J. Cunningham

**Affiliations:** Department of Anatomy and Neurobiology, College of Medicine, Drexel University, Philadelphia, Pennsylvania, United States of America; Center of Ophtalmology, Germany

## Abstract

There is increased interest in the contribution of secreted phospholipase A2 (sPLA2) enzymes to neurodegenerative diseases. Systemic treatment with the nonapeptide CHEC-9, a broad spectrum uncompetitive inhibitor of sPLA2, has been shown previously to inhibit neuron death and aspects of the inflammatory response in several models of neurodegeneration. A persistent question in studies of sPLA2 inhibitors, as for several other anti-inflammatory and neuroprotective compounds, is whether the cell protection is direct or due to slowing of the toxic aspects of the inflammatory response. To further explore this issue, we developed assays using SY5Y (neuronal cells) and HL-60 (monocytes) cell lines and examined the effects of sPLA2 inhibition on these homogeneous cell types *in vitro*. We found that the peptide inhibited sPLA2 enzyme activity in both SY5Y and HL-60 cultures. This inhibition provided direct protection to SY5Y neuronal cells and their processes in response to several forms of stress including exposure to conditioned medium from HL-60 cells. In cultures of HL-60 cells, sPLA2 inhibition had no effect on survival of the cells but attenuated their differentiation into macrophages, with regard to process development, phagocytic ability, and the expression of differentiation marker CD36, as well as the secretion of proinflammatory cytokines TNF-α and IL-6. These results suggest that sPLA2 enzyme activity organizes a cascade of changes comprising both cell degeneration and inflammation, processes that could theoretically operate independently during neurodegenerative conditions. The effectiveness of sPLA2 inhibitor CHEC-9 may be due to its ability to affect both processes in isolation. Testing potential anti-inflammatory/neuroprotective compounds with these human cell lines and their conditioned media may provide a useful screening tool prior to *in vivo* therapeutic applications.

## Introduction

Secreted phospholipases A2 (sPLA2) are several closely related enzymes with molecular masses of 13–20 kDa, belonging to a growing family of PLA2 enzymes (see review [1]). PLA2 family catalyzes the hydrolysis of glycerophospholipids at a single A2 bond, producing a free fatty acid and a lysophospholipid. The excessive hydrolysis of membrane phospholipids by activated sPLA2s can lead to the alteration of membrane function which can eventually lead to the functional failure of the membrane and cell death [2], [3]. Furthermore, the free fatty acids and lysophospholipid products of hydrolysis are precursors for bioactive pro-inflammatory mediators such as eicosanoids and platelet-activating factor (PAF). The sPLA2s in particular are attractive therapeutic targets because of their accessibility in the circulation and the fact that high levels of systemic enzyme activity characterize and contribute to most inflammatory disorders (see reviews [4], [5]), including many neurodegenerative diseases [6]–[8]. Recent experimental studies support this suggestion demonstrating sPLA2 are involved in nervous system trauma and autoimmune disorders [6], [9]–[11].

CHEC-9(CHEASAAQC) is a potent uncompetitive sPLA2 inhibitor that has been identified in our lab as an internal fragment of the survival promoting, anti-inflammatory polypeptide DSEP/Dermcidin/PIF [12]–[14]. CHEC-9 is considered “broad-spectrum” because it inhibits the diverse enzyme activities in the plasma of rats and humans (dominated by groups IIA and X sPLA2s), and inhibits the purified enzymes in groups I and III [9], [10], [14]. Since CHEC-9 is an uncompetitive inhibitor, it binds the enzyme substrate complex, so rather than strict sPLA2 isotype specificity, the inhibitor may prefer certain enzyme-substrate combinations [9]. Importantly, the anti-inflammatory and neuroprotective effects of sPLA2 inhibition by CHEC-9 have been documented for several *in vivo* models. For example, one subcutaneous injection of CHEC-9, 30–40 min after cerebral cortex lesions inhibited the appearance and activation of macrophages/microglia and protected cortical neurons [15]. Similar effects have now been reported for spinal cord and traumatic brain injury [16], [17]. In experimental autoimmune encephalomyelitis (EAE), a multiple sclerosis model, systemic treatment with CHEC-9 or with related uncompetitive inhibitor CHEC-7, inhibited microglia activation, demyelination and motor paralysis [9], [10]. A central question in these studies, and for the further development of sPLA2 and other inflammation-targeted therapeutics, is to determine the principal activity of these compounds, whether they protect cells by inhibiting toxic inflammatory responses, or by inhibiting cell death and thereby attenuating the inflammation. In the present study, we utilized homogenous human SY5Y and HL-60 cell lines. SY5Y neuroblastoma cells can be differentiated into cells with morphological and biochemical characteristics of mature neurons after stimulation with retinoic acid [18]. HL-60 leukemia cells can be differentiated into macrophages by treatment with phorbol 12-myristate 13-acetate (PMA) [19]. The results demonstrate that this sPLA2 inhibitor independently promotes neuronal cell survival, but not differentiation, and inhibits macrophage differentiation but not survival. Given the well known effects of sPLA2 enzymes on inflammation and cell survival, these dual effects are what might be expected for an efficient enzyme inhibitor and may explain the efficacy of the CHEC peptides when applied to *in vivo* models.

## Results

### Inhibition of sPLA2 enzyme activity by CHEC-9

**Figure 1:**
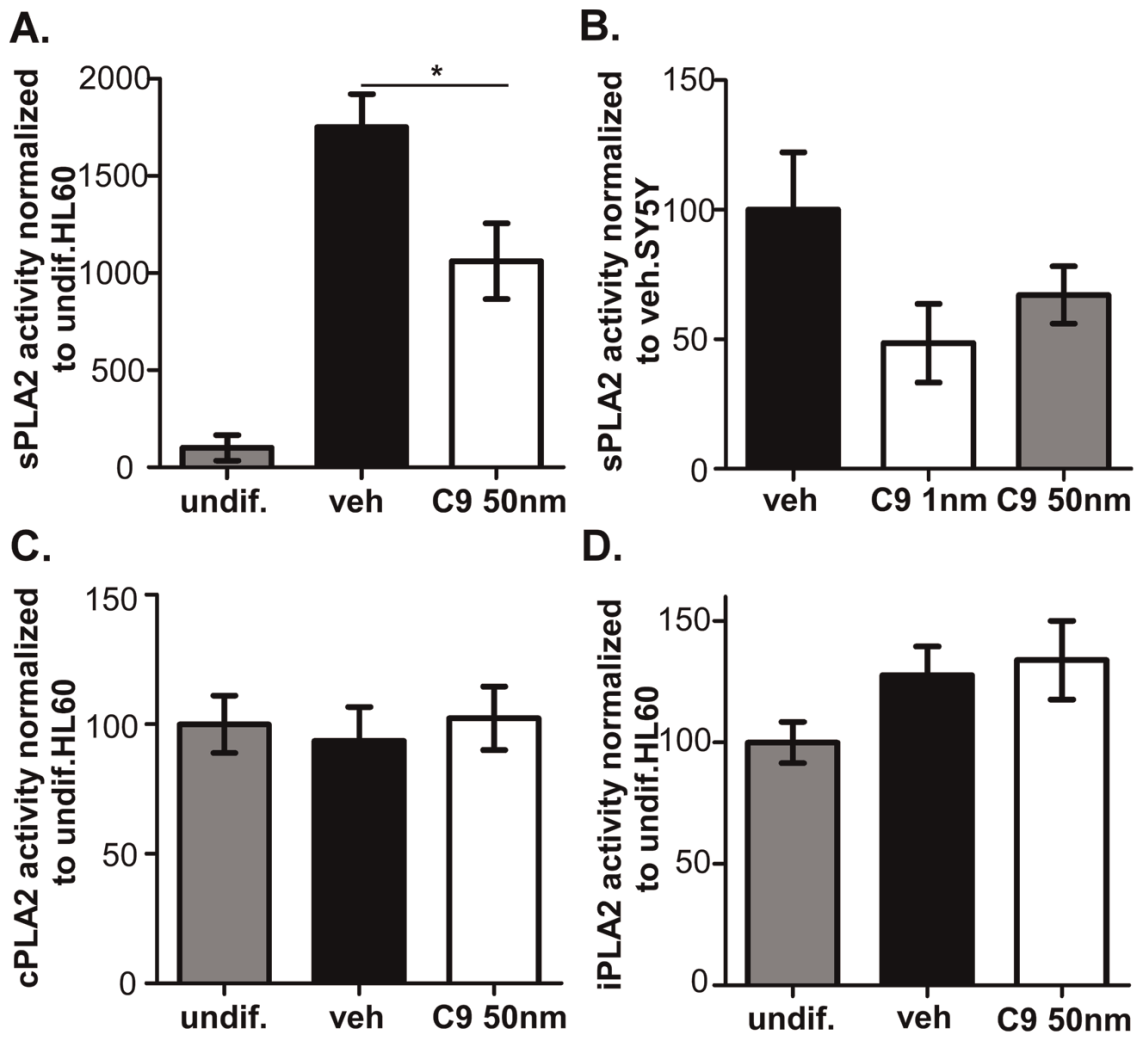
Measurement of PLA2 activity. (**A**) HL-60 cells were treated with 50 nM CHEC-9 or TBS vehicle for 4days with stimulation of PMA. The large increase of sPLA2 activity after differentiation was significantly attenuated by CHEC-9 treatment. (**B**) Differentiated SY5Y neuronal cells were subjected to medium change and serum deprivation for 2days. Compared with vehicle group, CHEC-9 treatments at 1 and 50 nM both reduced sPLA2 activity, but this change just missed significance (p = 0.07). (**C, D**) cPLA2 and iPLA2 activity of HL-60 cell homogenates. There was no difference between the undiffentiated and differntaited HL-60 cells. CHEC-9 treatment did not show inhibition of the cPLA2 or iPLA2 activities.

In order to confirm the CHEC-9 inhibition of enzyme activity, we measured sPLA2 hydrolysis in the media of SY5Y and HL-60 cells. Macrophage differentiation was accompanied by a dramatic increase in enzyme activity in both the vehicle (1750±171%) and CHEC-9 (1061±195%) groups, compared with the undifferentiated cells (100±66.6%, Fig. 1A). CHEC-9 treatment at the optimal concentration of 50 nM significantly reduced the sPLA2 activity in the medium after 4 days in culture (p = 0.03). In the SY5Y culture after two day's exposure to serum deprivation, similar reductions in sPLA2 activity were found with a single treatment of CHEC-9: 1 nM- 48.5±15.2%; 50 nM- 67.1±11.1%; vehicle- 100±22.2%, (Fig. 1B). For the medium of these cells however, individual values were much more variable and the effect just missed significance (p = 0.07).

Measurements of cPLA2 and iPLA2 activity in HL-60 cell homogenates revealed no significant differences between undifferentiated cells (cPLA2:100±11.1%; iPLA2 100±8.48%) and differentiated cells (cPLA2: 93±13.1%; iPLA2 127±11.9%). No inhibition of cPLA2 or iPLA2 activity was found by CHEC-9 treatment (cPLA2:102±12.2%; iPLA2 133±16.1%) (Fig. 1C, D), suggesting the selectivity of this peptide under present culturing conditions.

### CHEC-9 promoted the survival of SY5Y neurons and neuritic processes in response to medium change, serum deprivation and macrophage conditioned medium

After 4days of differentiation with retinoic acid, SY5Y cells exhibited their typical morphology of mature neurons with large round nuclei and 3–5 neuritic processes. The stress induced by medium change and serum deprivation led to a small loss of cells (13.7±4.6%) and a more pronounced loss of neuritic processes (40.2±7.4%). Dose-response analysis showed that CHEC-9 at 1 nM significantly increased cell and fiber numbers to near control values, consistent with previous studies on the parent polypeptides of CHEC-9 [12], [13].

**Figure 2:**
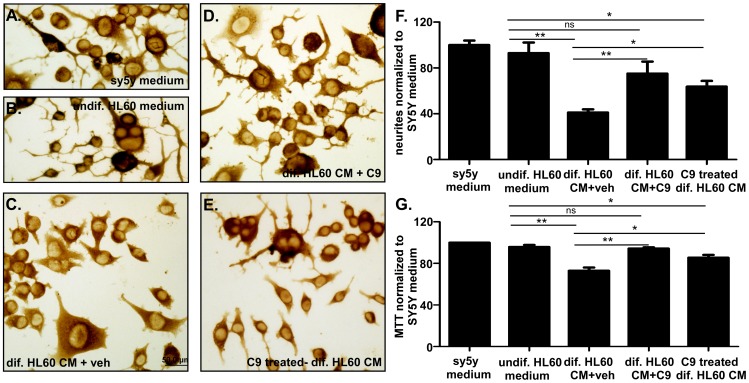
Protective effects of CHEC-9 on SY5Y cells. Retinoic acid differentiated SY5Y neuronal cells were subjected to different conditioned media for 72 hrs. **Cell process survival (A–F).** Long and elaborated processes were seen in cultures that were subjected to control media either from SY5Y cultures or undifferentiated HL-60 cultures (**A, B**). Dramatic degeneration of neuritic processes was found when SY5Y cells were subjected to medium from differentiated HL-60 cultures (**C**). These degenerative changes were reversed by CHEC-9 (50 nM, **D**). Medium from HL-60 cultures -pretreated with 50 nM CHEC-9 during differentiation, also preserved processes (**E**). (**F**) Quantification of the results. **Cell viability** (**G**). Both SY5Y conditioned medium and undifferentiated HL-60 did not affect SY5Y survival, whereas the medium from differentiated HL-60 cells significantly decreased viability. 50 nM CHEC-9 significantly restored the cell number to control level. Medium from HL-60 cultures-pretreated with 50 nM CHEC-9 during differentiation, also promoted cell survival. (* *p*<0.05, ** *p*<0.005).

When SY5Y cells were subjected to medium from differentiated HL-60 cells, there was also a significant loss of SY5Y cells by 28.9±3.04% (Fig. 2G) and loss of neuritic processes by 59.1±2.9% (Fig. 2C, F) compared with control media, obtained either from SY5Y culture (Fig. 2A) or undifferentiated HL-60 cells (Fig. 2B). Again, CHEC-9 treatment significantly improved the survival of cells and processes (restoring the former to near control level), but the effects required a higher concentration of 50 nM than that supporting cells subjected to oxidative stress (Fig. 2D, G). We also tested conditioned medium from HL-60 cultures that were treated with CHEC-9 during differentiation. The medium from these HL-60 cells also resulted in significant rescue of cells and processes compared to vehicle-treated HL-60 conditioned medium (Fig. 2F, G; also compare Fig. 2C to 2E).

In experiments to determine whether sPLA2 inhibition influence SY5Y cell differentiation, different concentrations of CHEC-9 were added into SY5Y along with retinoic acid. There was no effect on the differentiation of SY5Y cells in terms of the process development, and only a slight protective effect during differentiation (maximum of 7% increase at 50 nM of CHEC-9) compared with vehicle culture (data not shown).

### CHEC-9 treatment inhibited process development and expression of CD36 of HL-60 cells during differentiation without affecting viability

**Figure 3:**
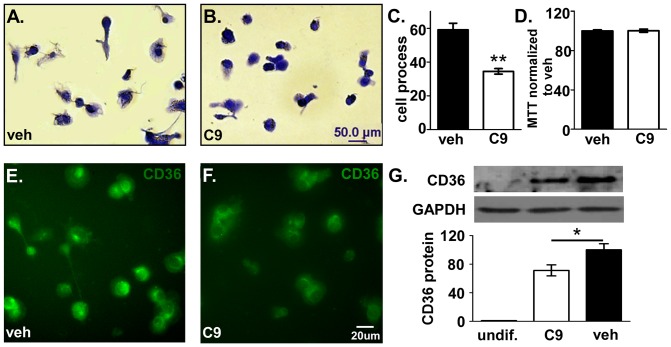
Inhibition of differentiation of HL-60 cells by CHEC-9. HL-60 cells were treated with 50 nM CHEC-9 or TBS vehicle for 4days with stimulation by PMA. HL-60 cells adhere to plate surface, flatten out and grow processes (**A**). CHEC-9 treatment decreased the number of cells with process (**B**). Quantification of Coommassie blue staining (**C**). CHEC-9 did not affect viability of the cells (**D**). Immunostaining for CD36 showed that the fluorescent intensity of CHEC-9 group was decreased compared with vehicle group (**E, F**). This observation was supported by western analysis (**G**). Quantification was by normalization to GAPDH bands. (* *p*<0.05, ** *p*<0.005).

We found that PMA inhibited cell proliferation and induced differentiation consistent with other studies using HL-60 cells as a model of human macrophage differentiation [20]. Differentiation includes cell adhesion spreading with process development, expression of differentiation markers and cytokines, as well as phagocytic activity, which are all characteristics of the macrophage phenotype [19]. For the present experiments, the optimal CHEC-9 concentration (50 nM, determined by dose response) was added to medium with PMA and the cells differentiated for 4 days. HL-60 cells adhered to the culture plates, spread out, and most grew processes (Fig. 3A). However, in the presence of CHEC-9, the number of cells with processes decreased (Fig. 3B). Instead there were large areas of small densely-stained HL-60 cells. Quantification of the cell number with process showed that CHEC-9 treatment significantly decreased the number of cells with processes compared with vehicle group (vehicle: 59±3.9%, CHEC-9: 34.4±1.6%, Fig. 3C). However, the total cell number of HL-60 cells in two groups was not different, at least as measured by the MTT assay, suggesting that CHEC-9 had no effects on the survival of HL-60 cells (vehicle: 100±1.1%; CHEC-9: 100.3±1.6%; Fig. 3D). Even when differentiated HL-60 cells were subjected to medium change and serum deprivation, where there was a loss of 33.2±3.6% of the cells, CHEC-9 did not show protective effect on the survival of these cells (data not shown).

Upregulation of a number of differentiation markers including CD36 have been documented as HL-60 cells differentiated into macrophages [21]. Given the influence of the sPLA2 inhibition on the morphology of HL-60 cells, we tested peptide effects on the expression levels of CD36, a marker associated with activation and phagocytosis. As shown in Figure 3 by immunostaining, the differentiated HL-60 cells were all CD36 positive in both the CHEC-9 and vehicle groups, but the fluorescent intensity was greatly decreased by sPLA2 inhibition (Fig. 3E, F). Western-blot results further confirmed the overall reduction of this protein (Fig. 3G). Four days stimulation by PMA increased the expression of CD36 in peptide and vehicle groups, but the intensity of CD36 bands was significantly attenuated to 71.2±7.1% by CHEC-9 treatment when compared with vehicle group (100±8.4%). (Fig. 3G)

### CHEC-9 inhibited phagocytic activity and cytokine levels of macrophages differentiated from HL-60 cells

**Figure 4:**
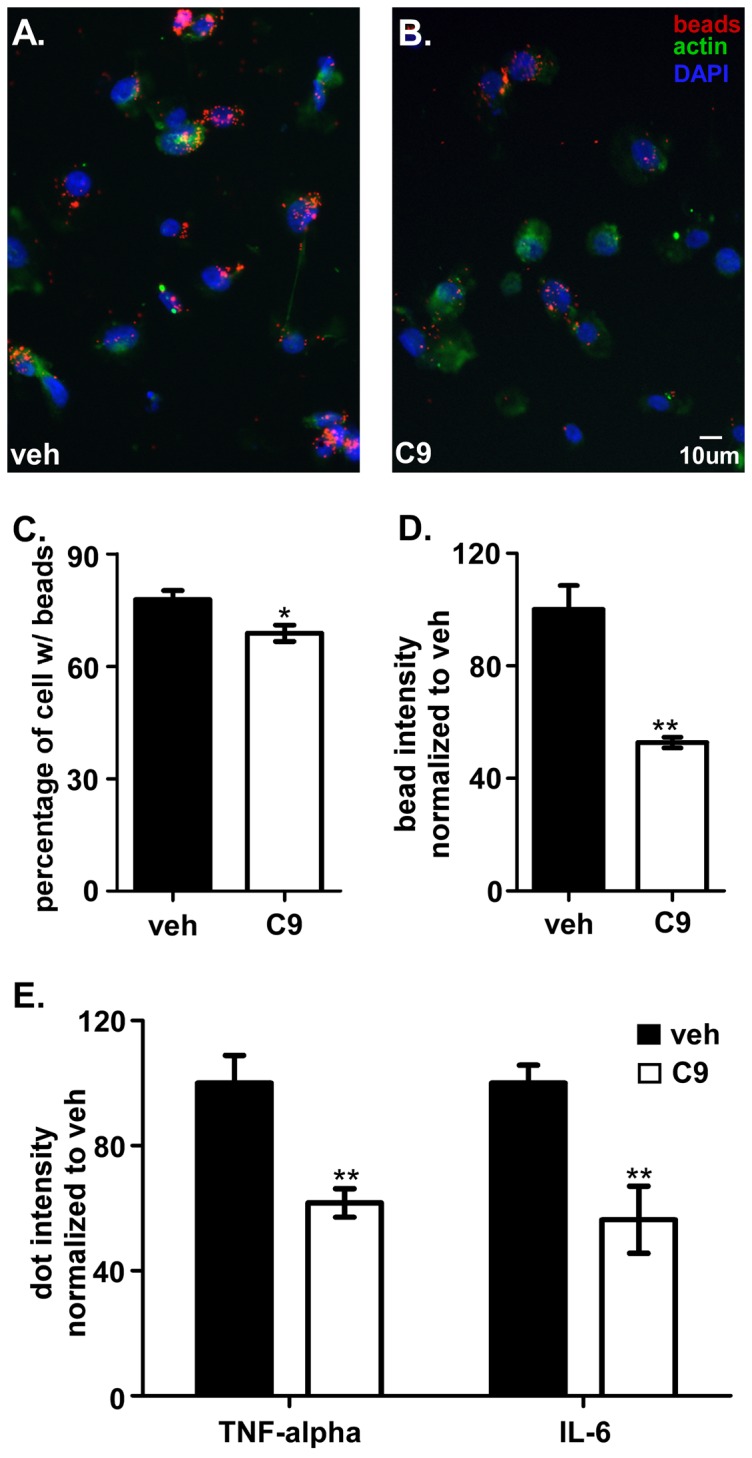
Phagocytosis activity and cytokine levels of macrophages differentiated from HL-60 cells. Most macrophages in vehicle group were labeled with red beads and the intensity of beads was high (**A**). Macrophages treated with CHEC-9 engulfed significantly fewer beads after 4 days differentiation and resulted in a lower percentage of cells containing beads. Quantification of the results (**C, D**). Decreased expression of TNF-α and IL-6 by 50–60% was found in the cell medium of CHEC-9 treated group (**E**). (** *p*<0.005).

Phagocytic activity is another characteristic of differentiated macrophages. It has been reported PMA activated HL-60 cells are capable of phagocytosis (including cannibalism) similar to activated native macrophages [22]. In order to test the influence of sPLA2 inhibition on this activity of the HL-60 cells, we utilized 0.5 µm (diameter) fluorescent latex beads. Previous studies have demonstrated the value of these for measurements of phagocytosis [23]. In our study, most of the HL-60 derived macrophages in the vehicle-treated group were labeled with red fluorescent beads after 24 hrs incubation (Fig. 4A). The intensity of single cell labeling, which is proportional to the number of beads engulfed by the cells was also high. Macrophage cultures that were treated with CHEC-9 had a slightly lower percentage of cells containing any beads, but individual cells contained about half as many beads as the vehicle treated group (Fig. 4D). These results suggested CHEC-9 has the most profound effects on the efficiency of phagocytosis, a result consistent with the peptide's effect on other aspects of differentiation.

During the differentiation of HL-60 cells into macrophages, the production of cytokines such as TNF-α and IL-6 has been documented as an early response to PMA-induced activation [24]. These cytokines are two of the toxic mediators that are released by macrophages in the inflammatory processes in various diseases including neurodegenerative disorders [25]. Given the interaction of sPLA2 with cytokines and the inhibition of HL-60 differentiation by CHEC-9, we measured the secretion of those two cytokines in the HL-60 medium. These studies were conducted using dot blots because of the relatively low total protein in the medium, and the attenuation of the specific cytokine signal during processing for western analysis. Our results showed that sPLA2 inhibition decreased HL-60 cell secretion of TNF-α to 61.7±4.53% and IL-6 to 56.3±10.7% when normalized to the vehicle groups (TNF-α: 100±8.86%; IL-6:100±5.78%, Fig. 4E).

## Discussion

The results confirmed the inhibition of sPLA2 activity by CHEC-9 in both SY5Y and HL-60 cultures with no detectable effects on the other major isoforms of PLA2 (cPLA2 and iPLA2). And this inhibition provided direct neuroprotective effects toward SY5Y neuronal cells in response to stressful stimuli induced by serum deprivation or macrophage conditioned medium. Under the conditions of cell stress used in these experiments, baseline cell loss was relatively mild and processes degradation was more pronounced. CHEC-9 inhibited both of these degenerative changes. In cultures of HL-60 cells, sPLA2 enzyme inhibition decreased the differentiation into macrophages induced by PMA without affecting cell viability. In this case, CHEC-9 treatment inhibited process development along with the expression of differentiation/phagocytosis marker CD36, phagocytic ability and the secretion of two prominent proinflammatory cytokines: TNF-α and IL-6. The reduction of toxicity of conditioned medium from CHEC-9 treated HL-60 cells further suggests that the peptide's influence on these cytokines contributes to the protection of neuronal cells exposed to macrophage conditioned medium. Therefore, this study suggests that sPLA2 inhibition has different and distinct effects on these neuronal and immune cell representatives, supporting the enzyme's leading roles in survival and inflammatory cascades that often characterize neurodegenerative disorders. The results further justify the view that sPLA2 enzymes would be a productive therapeutic target for these disorders [7]. We also suggest that these results explain the potent neuroprotective properties of CHEC-9 *in vivo* [9], [10], [14], [15], where the survival and anti-inflammatory effects of the peptide are likely to be independent and additive. Furthermore, this study also provides an *in vitro* testing system, using human cell lines to explore the cellular targets of the potential neuroprotective/anti-inflammatory therapies.

### sPLA2 inhibition via CHEC-9 promotes neuronal cell survival directly

In our previous study, treatment with a longer peptide(YP-30), or the over-expression of the full-length parent molecule DSEP (diffusible survival evasion peptide) in neuronal cells, provided remarkable survival promoting effects under conditions of stressful stimuli such as medium change and serum deprivation or even hydrogen peroxide exposure [12], [13]. CHEC-9 is an internal fragment of YP-30 and DSEP; and in this study it exerted a similar protective effect toward SY5Y neuronal cells against stressful stimuli. The inhibitory property of this peptide toward sPLA2 enzyme activity has been well studied in our previous work [14], [15]. The sPLA2 inhibition by this particular peptide sequence (CHEASAAQC) appeared to be highly specific since inversion of a single pair of amino acids (CHAESAAQC) eliminated the inhibitory effect [15]. The uncompetitive and broad-spectrum properties of this peptide may be advantageous with regard to *in vivo* effectiveness [14], [26]. In the present study, we confirmed the inhibition of sPLA2 enzyme activity in SY5Y cultures, and these results further support the suggestion that the sPLA2s are involved in direct oxidant destruction of neurons [27], [28]. Oxidative stress can activate sPLA2 enzymes which in turn hydrolyze the membrane phospholipids and impair the integrity of the membrane, all leading to the death of the cell [2], [3]. In addition, sPLA2 metabolism of phospholipids is a well-established source of reactive oxidative species (ROS) especially during the production of eicosanoids from arachidonic acid (AA). In metabolic processing of AA by cyclooxygenases (COX) and lipoxygenases (LOX), free radicals are produced as highly reactive intermediates or by-products such as superoxide radicals (O_2_^−^), or hydroxyl radicals(•OH) [29]. Additionally, the enzymatic activity of sPLA2 is directly involved in the generation of ROS, mainly due to the hydrolysis of mitochondria membrane phospholipids, causing the disruption of mitochondrial respiratory chain [30], [31]. Treatment with sPLA2 inhibitor CHEC-9 appears to interfere with the above processes explaining the survival promoting effects of this peptide. Interestingly, despite this survival-promoting activity in neuronal cells, the peptide did not show protection toward HL-60 cells. This result is consistent with previous studies that sPLA2-IIA had no toxic effects on guinea pig alveolar macrophages, human blood monocytes, HL-60 and U937 monocytic cell lines [32]–[34]. It may be that cells which produced significant quantities of proinflammatory and toxic mediators as scavengers and disinfectants are immune to the autocrine effects of these mediators.

### sPLA2 inhibition via CHEC-9 is also anti-inflammatory

In addition to reactive oxygen species, proinflammatory cytokines (such as TNF-α and IL-6), lytic enzymes (such as sPLA2), lipids mediators (such as lysophospholipids) are among the most potent inflammatory mediators that are released by activated immune cells after neural trauma or during the progression of CNS neurodegenerative disorders [35]–[37]. Although the principal function of these activated immune cells is the destruction of foreign organisms and phagocytosis of degenerating elements, activation of these cells to perform these functions also means collateral damages: the secretion of toxic mediators that can kill non-degenerating bystander cells and axons of passage, and thereby exaggerate functional deficits that result from lesions [38], [39]. In this study, we inhibited sPLA2 enzyme activity and the secretion of proinflammatory mediators from HL-60 cells; protected SY5Y cells from degeneration after exposure to the macrophage conditioned medium. This medium is much less toxic when the cells are pretreated with CHEC-9, although it is possible that CHEC-9 that was originally added to HL-60 cells was carried over to SY5Y culture when we transferred the conditioned medium. However, based on the finding of the strong influence of CHEC-9 on HL-60 differentiation, we conclude that indirect effects via inhibition of HL-60 cell functions and secretions must at least contribute to the protective activity of the peptides observed after medium transfer. In addition, there is abundant experimental support for interaction of cytokines with sPLA2 enzymes including the existence of a positive feedback loop in several cell types. For example, sPLA2-IIA mRNA and protein expression and PLA2 activity were all increased after exposure of astrocytes cultures to proinflammatory cytokines (TNF-α, interleukin-1β and interferon γ) [11], [40]. The bioactive downstream PLA2 products, such as linoleic acid, lysophosphatidylcholine and prostaglandin E2 contributed to the inflammatory cascade by upregulation of inflammatory cytokines such as TNF-α and IL-6 in macrophages [41]; prostaglandin E2 and TNF-α also have synergic effects on promoting the activation of transcription factor NF kappaB [42]. The activation of NF kappaB by TNF-α in keratinocytes is strongly reduced by a specific sPLA2 inhibitor. This result suggests that the downstream effects of TNF-α depend at least in part on sPLA2 [43].

The present results also support the suggestion that sPLA2 enzymes contribute to macrophage differentiation and activity. Exogenous snake venom PLA2 significantly potentiated the differentiation of HL-60 cells to macrophages with the presence of PMA [44]. Similar effects were also observed with the products of PLA2 hydrolysis activity: lysophosphatidylcholine and free fatty acids such as linoleic acid [21]. These findings, together with our results, suggest that activation of sPLA2 may be intimately related to the signal transduction pathway during HL-60 differentiation. As shown in the present study, the production and/or release of cytokine TNF-α was attenuated as a result of sPLA2 inhibition. The decreased TNF-α level also helps to explain CHEC-9 actions because it has been reported that TNF-α regulated macrophage differentiation of HL-60 cells in an autocrine manner [45]. Another crucial function of macrophages is the capacity to capture exogenous antigens, that is, phagocytic activity. We found that sPLA2 inhibition by CHEC-9 significantly altered the processes of phagocytosis and the expression of one phagocytic receptor CD36 on HL-60 cells. These findings are consistent with previous studies. When sPLA2-IIa was added to THP cells–another human monocyte cell line, their endocytic capacity was significantly enhanced [32]. In addition, sPLA2-V was found to directly regulate phagosome formation in mouse macrophages [33], [34]. In addition to these previous studies, the present work adds another line of evidence that strongly supports the crucial roles of sPLA2 in innate immunity and inflammation.

SY5Y cells and HL-60 cells are considered good *in vitro* models for the study of neurodegeneration and monocyte/macrophage differentiation respectively [18], [19]. In the present study, using isolated cells, we tested the effects of the peptide on the two major representatives of the neurodegeneration: neuronal and immune cell. Then, by using conditioned media, one mode of neuronal-inflammatory cell interactions was investigated. It is possible that this method can be adapted for high-throughput screening of drugs targeting a variety of neurodegenerative disorders.

In summary, we designed *in vitro* tests to indentify the cellular targets of sPLA2 enzymes, and with this testing system, we provided experimental evidence that sPLA2 inhibitor CHEC-9 had differential effects on neuronal and immune cells. In addition to the direct protection of neuronal cells, this peptide can independently inhibit immune cell responses and therefore also protect the cells indirectly. Since there is lack of effective treatments for most neurological disorders, the ability to explore these different modes of neuronal protection *in vitro* may be of great interest for the design of such therapies.

## Materials and Methods

### Peptide preparation

CHEC-9 (CHEASAAQC) was synthesized as a linear peptide by Celtek, Inc. The peptide was cross-linked for optimal stability and effectiveness in tris-buffered saline (TBS, pH 7.8) at 0.25 mg/ml overnight. Free sulfhydryls were measured using Ellman's reagent and were undetectable after cross-linking, which was further verified by electrospray mass spectrometry as in previous studies [14].

### Cell culture and differentiation

The human SY5Y neuroblastoma cell line (American Type Culture Collection, Manassas, VA) [18] and human HL-60 myeloid leukemia cell line (American Type Culture Collection, Manassas, VA) [19] were cultured in Dulbecco's modified Eagle's medium (DMEM) and RPMI 1640 medium (Mediatech, Manassas, VA) respectively, supplemented with 10% fetal bovine serum (FBS), 2 mM L-glutamine and antibiotics (penicillin 100 U/ml and streptomycin 100 g/ml) at 37°C in a saturated humidity atmosphere containing 95% air and 5% CO2. Treatment and medium change were every other day.

SY5Y cells were plated at the density of 1×10^5^ cells/ml and were differentiated into neuronal phenotype with 15 µM all-trans-retinoic acid (Sigma, St. Louis, MO) for 4 days. Differentiated SY5Y cells were subjected to rapid change to serum free medium or exposed to conditioned medium obtained from differentiated HL-60 cultures. Medium from undifferentiated HL-60 cultures or SY5Y cultures served as controls. Forty-eight to seventy-two hours later, cell and neurite survival were examined. In some experiment, SY5Y cells were treated with CHEC-9 or TBS vehicle during the differentiation period.

HL-60 cells were cultured at a density of 1×10^6^ cells/ml and differentiated into macrophages by treatment with 20 nM phorbol 12-myristate 13-acetate (PMA). TBS vehicle or CHEC-9 was added to HL-60 cells to study the effects of sPLA2 inhibition in different aspects of macrophage differentiation. In other experiments designed to mimic treatment of SY5Y cells, differentiated HL-60 cells were treated with CHEC-9 or vehicle after medium change and serum deprivation. For cell viability assay, cells were cultured in 96-well plates. For the analysis of neurite survival/growth, CD36 marker expression and cell morphology, cells were plated in eight-well Lab-Tek chamber plastic slides (Thermo, Rochester, NY). All the plates and slides were previously coated with poly-L-lysine.

### PLA2 enzyme activity assay

Culture media from SY5Y and HL-60 cells were tested for sPLA2 enzyme activity by procedures similar to those described [10]. One-hundred microliter of cell-free medium was reacted with 200 µM 1,2-bis (heptanoylthio) glycerol-phosphocholine in a reaction buffer consisting of 50 mM tris, 0.1 M NaCl (pH = 7.4), 1 mM DTNB (Ellman's reagent). The reaction was initiated by the addition of CaCl2 (final concentration 2.5 mM). Reaction rates were determined with a Deltasoft (Princeton, NJ) supported ELX 808 reader (Biotek, Burlington, VT) and the rate of product formation was measured for 40 minute and calculated relative to control conditions. Cell homogenates from HL-60 cultures were tested for cPLA2 activity with minor modifications of manufactures suggested protocol(Cayman chemical, Ann Arbor, MI) and iPLA2 activity by procedures previously described [41]. Briefly, the cells were collected using cell scraper, homogenized and centrifuged at 20 000 g for 30 min at 4°C. The sPLA2 activity was removed from those samples using membrane filter with a molecular weight cut-off of 30,000 (Millipore, Danvers, MA). Fifty microliter of cell-free supernatant was reacted with 300 µM 2-deoxy-2-thio arachidonoyl glycerol-phosphocholine in the same buffer described above. CaCl2 was added to cPLA2 assays in the presence of iPLA2 inhibitor bromoenol lactone, whereas iPLA2 assay was calcium free. The overall reaction was determined as described above, and calculated relative to the undifferentiated HL-60 cultures.

### Cell viability assay

Cell viability assays were carried out using the MTT assay kit (Life Technologies, Grand Island, NY) according to manufacturer's instructions. A MTT standard curve was also obtained to make sure the measurements in those experiments fall into the linear range. Values obtained in different conditions were normalized to control cultures.

### Whole cell staining and neuritic process analysis

TUJ1 staining was carried out to examine the survival of neurites in SY5Y cells. Briefly, the cells were fixed for 20 min in buffered 2% paraformaldehyde and processed for immunostaining with TUJ1 primary antibody to neuronal specific tubulin (isotype III, Covance, Princeton, NJ) and HRP conjugated secondary antibody. Systematically defined fields were photographed and neurites were quantified by counting the number of fibers that intersect the grid lines which were overlaid in ImageJ.

After 4 days of differentiation, HL-60 cells in TBS vehicle or CHEC-9 groups were fixed with 2% paraformaldehyde and stained with Coommassie blue solution (0.2% Brilliant Blue in 20% methanol, 0.5% acetic acid). The percentage of cells with process was calculated in three fields of each well. For this and all other *in vitro* experiments, at least two independent sets of cell cultures were examined by experimentally blind observers and each experimental condition was analyzed in quadruplicate cultures.

### CD36 immunostaining and western blot analysis

HL-60 cells were fixed and processed for staining with antibody to macrophage differentiation marker CD36 (Santa Cruz Biotech, Santa Cruz, CA) and FITC- conjugated secondary antibody. Western-blots from lysed and homogenized HL-60 cells were prepared as described previously [46], [47], using CD36 and GAPDH antibodies. Undifferentiated HL-60 cells served as negative control. Thirty microgram of protein were subjected to Tris–HCl SDS–PAGE and transferred to nitrocellulose membrane (Bio-Rad Laboratories, Hercules, CA). After incubation with HRP-conjugated secondary antibody, the membranes were processed for ECL immunodetection and whole band analysis was conducted with Image J (NIH). Band intensities were normalized to GAPDH loading control. Samples were derived from at least three different cultures and, each sample was analyzed 3–4 times with peptide-treated and control treated samples run side by side.

### Measurement of TNF-α and IL-6

Cell-free medium of HL-60 cells in different groups was collected and tested using dot-blotting. Briefly, the protein concentration of those samples was measured and 1 µl of (4 µg/µl, 2 µg/µl or 1 µg/µl) samples were dotted onto a nitrocellulose membrane and air-dried for two hours. The membranes were blocked and stained with antibodies to TNF-α and IL-6 (Santa Cruz Biotech, Santa Cruz, CA) and the protein dots were detected with ECL. Negative control was incubated with secondary antibody only to detect non-specific signals. The dots densities were analyzed as above.

### Phagocytosis assay

Differentiated HL-60 cells were incubated for 24 hours with sterilized 0.5 µm (diameter) fluorescent latex beads (Sigma, St. Louis, MO) at the concentration of 6×10^6^/well. Thereafter, the cells were fixed, reacted with a β-actin antibody (Sigma, St. Louis, MO) define the cell outline and mounted with DAPI-containing medium. The phagocytic ability of cells were analyzed at least three fields of each culture by calculating the percentage of bead positive cells and the intensity of beads in individual cells using ImageJ.

### Statistical analysis

The data were expressed as mean ± SEM. Statistical significance between two groups was assessed with an analysis of variance followed by Student's t-test. Statistical significance between multiple groups was performed using a one-way analysis of variance (ANOVA). When ANOVA showed a significant difference, an LSD multiple comparisons post-hoc test was performed. A value of p<0.05 was considered significant.
